# A Coexisting Adjacent Intradural and Extradural Spinal Cavernoma: A Rare Case

**DOI:** 10.7759/cureus.72685

**Published:** 2024-10-30

**Authors:** Kevin Joseph Jacob, Omar Walid Rasheed, Ahmed Abdelaal, K Joshi George

**Affiliations:** 1 Department of Trauma and Orthopaedics, Worcestershire Acute Hospitals NHS Trust, Worcester, GBR; 2 Department of Neurosurgery, Salford Royal NHS Foundation Trust, Manchester, GBR

**Keywords:** babinski, cavernoma, endothelial, extramedullary, intramedullary

## Abstract

A 34-year-old female patient, with no comorbidities, presented with complaints of upper back pain across the shoulders, with altered sensation on the left side from trunk to lower limb, which was associated with reduced motor function and an acute symptom of urinary retention. On examination, there was reduced power in the left lower limb, reduced anal tone, a positive Babinski sign bilaterally, and reduced sensation in the perianal region. Serial magnetic resonance imaging (MRI) scans were conducted, where initially an upper thoracic lesion suggestive of an intramedullary cavernoma was found, and nearly a decade later, an adjacent extradural lesion causing cord compression was found incidentally through a surgical procedure. The extradural lesion was removed, and the histology confirmed a cavernoma.

In our report, we try to explain a rare case of a patient with co-existing extradural and intradural cavernomas within the spinal cord at different levels. If the patient is symptomatic, a detailed workup plan, along with diagnostic modalities, is needed for further management.

## Introduction

A cavernoma is a sinusoidal vascular malformation found predominantly in the brain and rarely in the spine. It is also called a cavernous angioma, cavernous haemangioma, or cavernous malformation. The incidence of spinal cavernomas is around 5% to 16% of all spinal vascular malformations, according to a few studies; other than that, not many have been proven [1,2].

Patients may present with various symptoms, ranging from being asymptomatic to acute or chronic progressive myelopathy, depending on the severity of spinal cord compression. The diagnostic imaging of choice for spinal cavernous malformations is magnetic resonance imaging (MRI) [3].

Spinal cavernous malformations are histologically identical to an intracranial cavernoma, which is characterized by lobulated, dilated sinusoidal channels of dense, capillary-shaped vessels. In terms of gross appearance, they are well-circumscribed lesions that appear like a mulberry [4].

Here, we describe a case in which a patient has coexisting adjacent intradural and extradural cavernomas of the spine, which is a rare occurrence. There are no previously documented cases in the literature.

Formal consent was not needed, as personal information has not been disclosed publicly. A discussion was held with the patient about this publication, and she was supportive of her case being published to educate other health professionals about this very rare condition.

## Case presentation

A 34-year-old woman with no comorbidities presented to the Emergency Department with a three-day history of upper back pain across the shoulders, accompanied by altered sensation on the left side of the body, from the trunk radiating down to the left lower limb. This was associated with loss of motor function affecting the same lower limb. She also had a one-day history of urinary retention, with the absence of bowel or bladder incontinence.

Examination

On neurologic examination, it was found that power in the left lower limb was 4/5 throughout and intact in the remaining limbs, with the Babinski sign being positive bilaterally. Otherwise, other examination findings, such as coordination, tone, and reflexes, were intact across both upper and lower limbs. At the level of the T5 dermatome and below - sparing the right lower limb - there was altered sensation bilaterally and in the perianal region. The altered sensation manifested as a 'tingling and burning' feeling on palpation, and anal tone was found to be reduced. However, the patient passed urine spontaneously three to four hours later, confirming she was able to feel the voluntary passage of urine.

Progression

The patient's motor function in the lower limbs deteriorated the following day. A dense worsening of weakness was noted in the left lower limb, with only the ankle maintaining movement, exhibiting a power of 2/5 (dorsiflexion and plantar flexion). The previously unaffected right lower limb demonstrated a significant reduction in power, now measuring 3/5 throughout.

**Figure 1:**
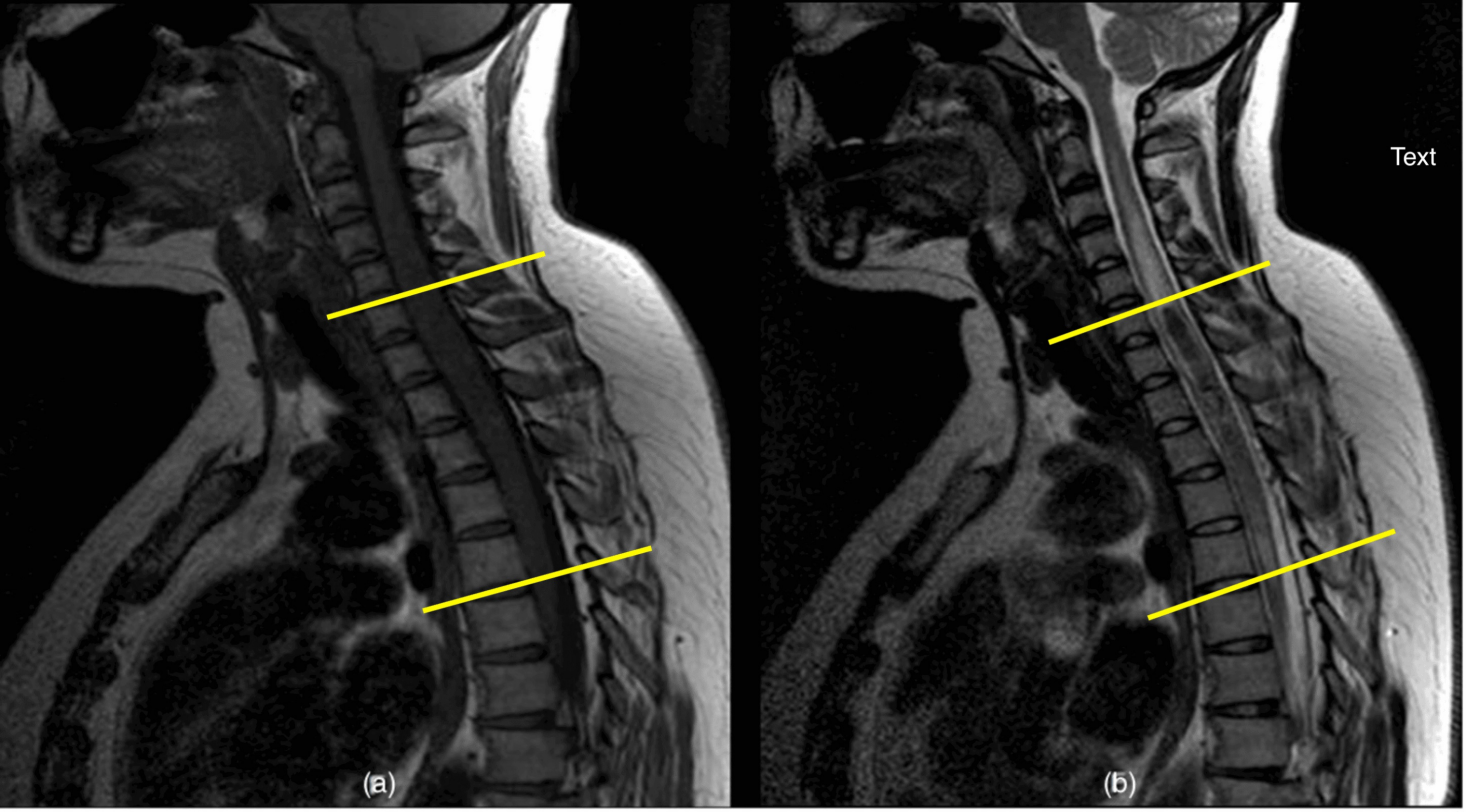
T1 and T2 sequenced MRI images (a) T1 sequenced and (b) T2 sequenced MRI of the sagittal section of the whole spine showing a lesion extending from level C6 down to T4 with associated oedema (described by yellow lines) MRI: Magnetic resonance imaging

An emergency MRI scan of the spine was planned immediately due to the significance of clinical worsening (one day after the onset of initial symptoms). In recognition of the altered sensation from the trunk down, a T2 scout view of the whole spine was chosen. Imaging (Figure 1) revealed a lesion within the spinal cord extending from the level of C6 down to T4, with associated oedema. Low T2 signals were noted in conjunction with increased signals on T1-weighted sequencing. Differential diagnoses included haemorrhage into a mass lesion and vascular malformation. There was a widening of the exit foramen at T2/T3, and posterior scalloping of the T2 vertebral body made the former more likely. 

**Figure 2:**
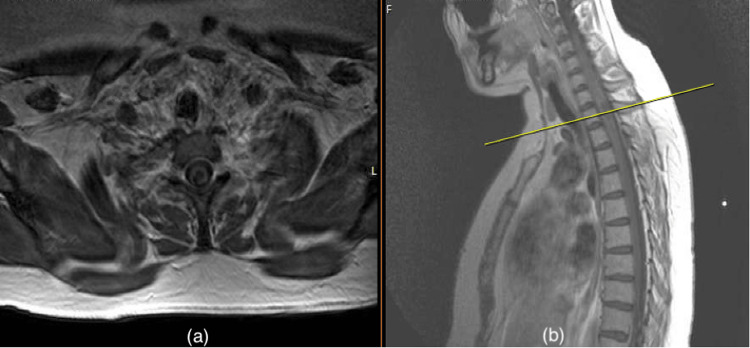
T2 sequenced MRI images T2 sequenced of MRI (a) axial section and (b) sagittal section of the whole spine showing an intrinsic intramedullary vascular lesion from level C6 down to T2 (as marked by the yellow line) MRI: Magnetic resonance imaging

The patient was recalled for a post-contrast MRI study (Figure 2). It demonstrated an abnormal, irregular enhancement from C5/C6 down to the level of T2, where serpiginous enhancement was also reported. This imaging was deemed suggestive of an intrinsic intramedullary vascular lesion.

As part of the medical treatment, the patient was started on dexamethasone at 4 mg twice a day (weaned to 2 mg twice a day around a week later). A multidisciplinary team (MDT) meeting was conducted to discuss the course of this case, where an opinion was made in favour of differentials such as bleeding into a tumour, cavernoma, and vascular malformation. Since the patient was neurologically stable and manageable, conservative management was chosen, with a repeat MRI of the spine in four to six weeks (to allow for clearance of blood). The patient was admitted to a neurorehabilitation ward for a short period of monitoring.

**Figure 3:**
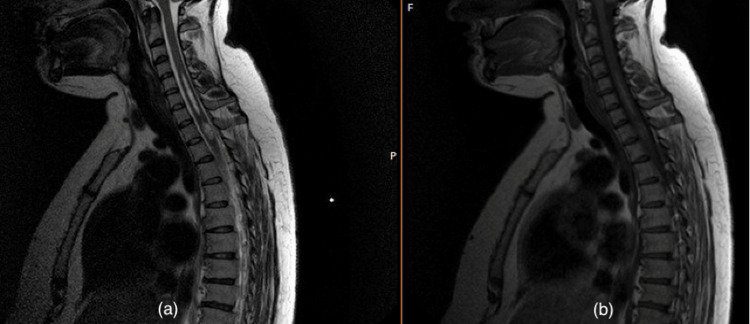
T2 sequenced and T1 sequenced MRI images (a) T2 sequenced and (b) T1 sequenced MRI of the sagittal section of the whole spine showing improvement in appearance from previously noted hematoma MRI: Magnetic resonance imaging

Over the next few weeks and months, the patient improved steadily until she could walk with a stick, with the need for intermittent self-catheterization. She made good, steady weekly progress with the assistance of the physiotherapy team. An interval scan (Figure 3) was performed during this process, where a significant improvement in overall appearance was observed. The previously noted hematoma had largely resolved, which was a breakthrough in moving toward a diagnosis. Due to the absence of abnormal vascular enhancement or flow voids, cavernoma of the spinal cord became the top differential. An MRI of the brain was performed to rule out an associated brain cavernoma, which was negative. After considering the improving neurological condition and low risk of bleeding, along with the potential risks, conservative management was once again chosen.

From then onwards, the patient had annual outpatient reviews under the Neurosurgery Department. She continued to make a functional recovery over the following years where mobility became possible with a walking stick. However, bladder function still remained impaired.

**Figure 4:**
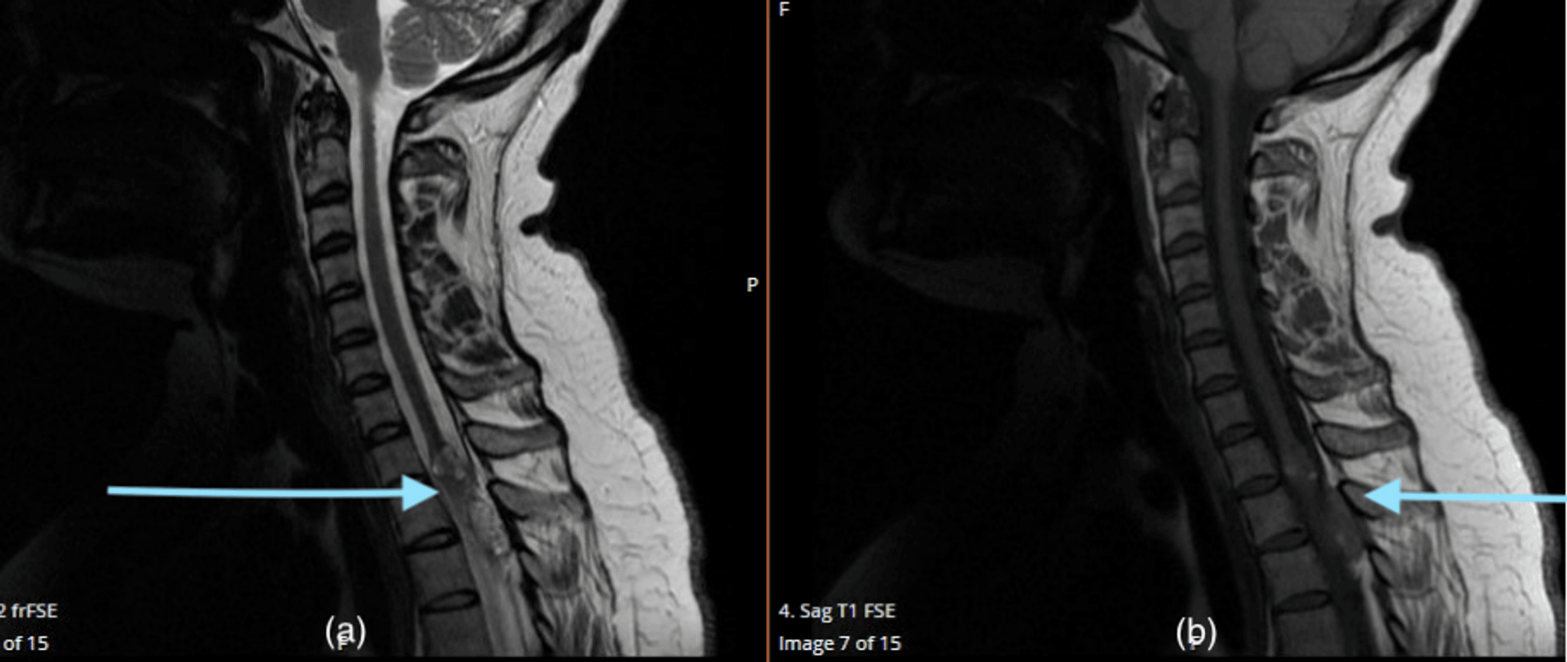
T2 sequenced and T1 sequenced MRI images (a) T2 sequenced and (b) T1 sequenced MRI of the sagittal section of the whole spine showing signs of cavernoma with evidence of previous bleeding (marked with blue arrows) MRI: Magnetic resonance imaging

**Figure 5:**
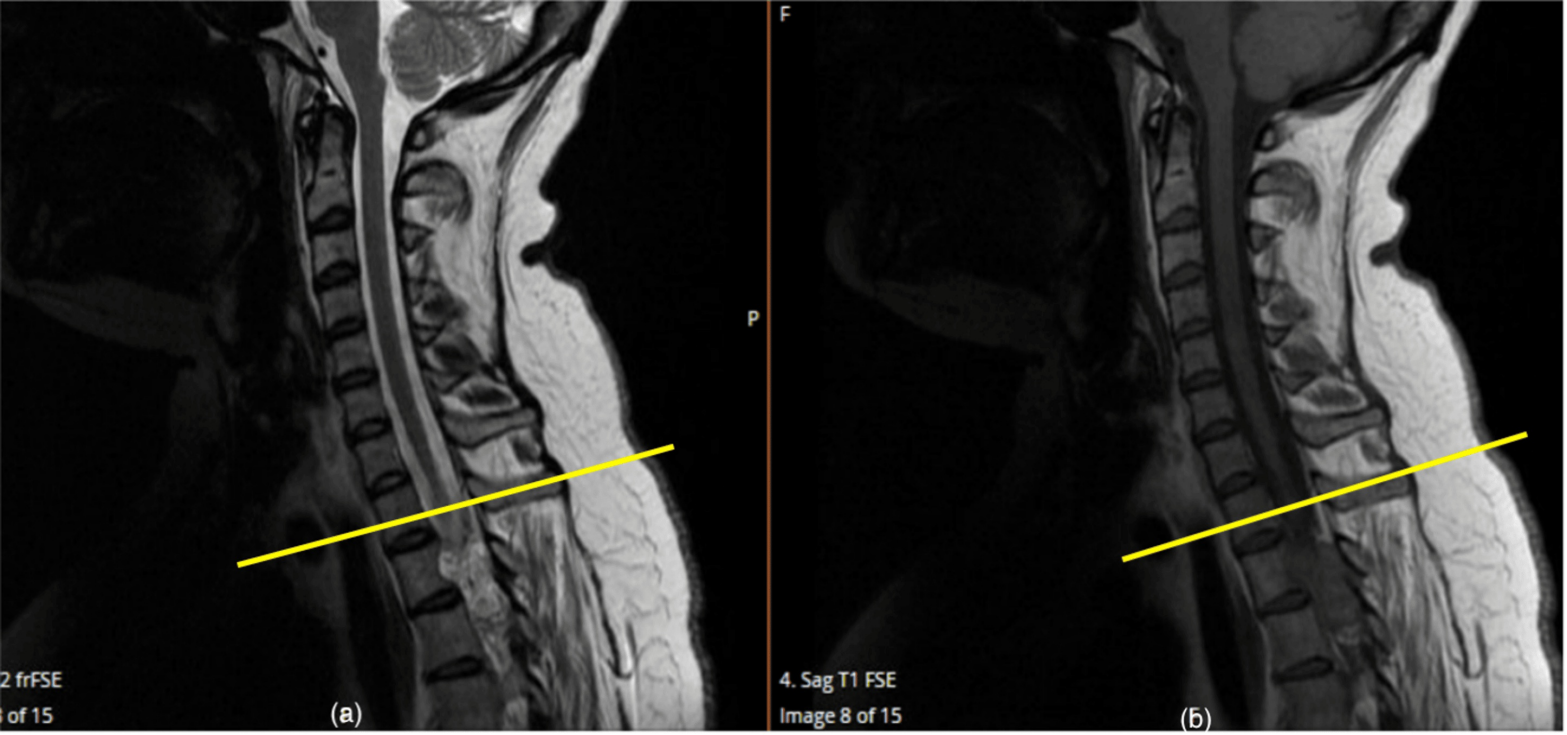
T2 sequenced and T1 sequenced MRI images (a) T2 sequenced and (b) T1 sequenced MRI of the sagittal section of the whole spine showing signs of cavernoma extending inferiorly to T2/T3 exit foramen - likely causing compression of T2 nerve root (marked below the yellow arrow lines) MRI: Magnetic resonance imaging

A follow-up MRI scan of the spinal cord was arranged 11 years after the patient’s initial presentation as a clinic appointment. The patient reported a new feeling of 'pins and needles' over the neck, along with pain in both arms. The imaging revealed a complex lesion with exophytic components arising from the T1 to T3 levels, characterized by a predominance of high T2 signals and more discrete areas of high T1 signalling. These were reported as clear signs of cavernoma in the superior components of the lesion, with evidence of previous bleeding present. A large exophytic component of the lesion was noted to extend inferiorly to the T2/T3 exit foramen, likely compressing the T2 nerve root (Figures 4-5). 

The patient’s neurology around the time of this latest follow-up scan was determined to be unlikely to have changed from the patient’s most recent baseline: a power of 4/5 throughout the right leg, 3/5 throughout the left leg, brisk lower limb reflexes, and intact neurology of the upper limbs (except for 'clawing of the fingers,' reported by the patient to occur during the night and remedied with the use of a splint). However, the patient remained in need of intermittent self-catheterization, as even regular Botox injections to the neck of the bladder did not yield much benefit. The patient’s independent mobility (without a walking stick) was limited to a few steps due to a spastic and unsteady gait.

With regard to the change in clinical scenario, the patient was planned for an MDT discussion, where a repeat MRI scan was also scheduled. However, a month later, they presented to the Emergency Department with a reported loss of sensation in the lower limbs. A neurological examination revealed reduced sensation from the L1 dermatome downwards bilaterally, as well as an acute onset of thoracic pain. Fortunately, the patient was found to be neurologically at baseline. The patient was admitted and booked for an MRI scan of the whole spine, which revealed an interval increase in the size of the inferior components (T2/T3) of the lesion, presumably a cavernoma, with an increased mass effect on the spinal cord.

Subsequently, a surgical procedure of right T2/T3 hemilaminectomy and debulking of the extradural components of the lesion was planned. Intraoperatively, a highly vascular extradural tumour was observed to extend to the right T2/T3 foramen, along with frontal scalloping of the bone and foraminal widening. Hence, debulking was performed using bipolar forceps and rongeurs. The specimen was sent for histologic analysis, which was later determined and confirmed to be a cavernoma. It consisted of dense fibrous connective tissue with diathermy, containing abnormal vascular channels with thick hyalinized walls. There were aggregates of hemosiderin-laden macrophages and chronic inflammation. The adipose tissue at the edge of the lesion showed myxoid changes and focal chronic inflammation. A repeat MRI scan of the spine was performed following the operation; this showed a complete removal of the extradural tumour (the lesion) at T2/T3.

Following the operation, the patient’s thoracic pain had resolved. Neurologically, there were no significant changes, and the patient was found to be similar to her condition before the procedure.

The patient was discussed in a neurovascular multidisciplinary meeting. Owing to the radiological appearance of the remaining intradural lesion, it was suggested that it was quiescent (with no signal change to the adjacent cord) and that it had not bled. The likelihood of bleeding from the lesion over the next five years was estimated to be around 20%. In view of the location of the intradural lesion, a risk of permanent harm to the dorsal columns and paralysis was identified. Conservative management was considered the best option, along with regular scheduled outpatient follow-ups.

## Discussion

Cavernomas, due to their abnormal location, can cause issues with the structures in the spinal cord, leading to weakness or numbness in the limbs, along with positive examination findings such as abnormal reflexes. The common presenting symptoms include sensory or motor deficits, radiculopathy, myelopathy, bowel or bladder incontinence, and back pain in specific areas at different levels. Spinal cavernomas are diagnosed based on the symptoms of the patients but can also be incidental findings on radiological scans.

It is said that around 10% of cases present with an acute onset of symptoms due to subarachnoid haemorrhage or thrombosis, which may bleed into the spinal cord, causing sudden onset of these symptoms [5]. Every year, the risk of bleeding increases by about 2% to 3% for patients with a cavernoma greater than 1 cm in diameter [6]. Symptomatic patients, as well as those with a previous history of bleeding, have significantly higher rates of recurrence of bleeding (approximately 9% to 10%) [3].

Spinal cavernomas are rare, making up only 5% of spinal vascular malformations [7]. However, intradural extramedullary cavernomas are even rarer, with about 40 case reports available, and a total of only 40 previously described. They are usually located in the cervical and thoracic regions of the spinal cord, with the incidence of thoracic cavernomas (46%) being the most common, followed by cervical (38%), cervicothoracic (8%), and conus (8%), which is the least common [8].

From 40 case reports, only four cases involved the upper thoracic spine [7,9]. This condition can be sporadic or familial, where individuals with single lesions who have unaffected relatives are most likely to have a sporadic disease, unlike those with multiple lesions, which indicate a familial type due to mutations in the gene [10].

Cavernomas are most commonly present between the third and sixth decades of life, with most cases occurring around 40 years of age [11-13]. However, they can vary in age from 2 to 80 years old. In terms of gender differentiation, 70% are reported to be female, according to a study [14]. Additionally, our case report involves a female patient.

There are cases reported in the literature where both intradural (extramedullary or intramedullary) and extradural cavernoma components exist, but not separately in the same person. A literature review revealed two case reports in which a spinal cavernous haemangioma had both intradural and extradural growth (i.e., through the dura mater). One of them showed symptoms of Brown-Sequard syndrome [15]. We found no cases where coexisting intradural and extradural spinal cavernomas are present simultaneously in the same patient. To the best of our knowledge, this is the first such case to be reported. Our case illustrates that when both an intramedullary and extradural cavernoma are present, removing the symptomatic extradural component only is an effective and safe management strategy, as there has been no recurrence of the extradural cavernoma and the patient did not suffer any postoperative neurologic deficit.

Prognosis

Spinal cavernomas are a very rare entity among spinal lesions, as mentioned, and must be considered a potential cause of subarachnoid haemorrhage. Therefore, an extended diagnostic workup is mandatory to prevent recurrent bleeding and permanent neurological deficits, which can lead to severe consequences, including stroke and even death [1]. The treatment for a spinal cavernoma is conservative for asymptomatic patients; however, surgical resection is preferred if symptoms of spinal root compression, sudden onset myelopathy, or progressive neurological deficits appear. This can be detected through useful modalities like MRI and histological examinations to confirm the diagnosis [16].

Differential diagnosis

The characteristics of spinal cavernous angioma on contrast-enhanced MRI include small size, eccentric location, minimal enhancement with contrast, and absence of oedema, compared to haemorrhagic ependymomas, which have larger size, central location, strong enhancement with contrast, and presence of oedema. Both have almost completely different radiological appearances [3]. They have a characteristic popcorn appearance on the MRI T2 sequence and are angiographically occult.

## Conclusions

In our report, we explain a rare case of a patient with coexisting extradural and intradural cavernomas within the spinal cord at different levels. If the patient is symptomatic, a detailed workup plan with diagnostic modalities is needed for further management.
